# Supplementary material for: Investigation of Gastrointestinal Toxicities Associated with Concurrent Abdominal Radiation Therapy and the Tyrosine Kinase Inhibitor Sunitinib in a Mouse Model
Source: Int J Mol Sci. 2024 Feb 2;25(3):1838. doi: 10.3390/ijms25031838 (PMC10855812; doi:10.3390/ijms25031838)
Supplement: Supplementary file 1 [file ijms-25-01838-s001.zip › ijms-2812678-supplementary.pdf]

International Journal of Molecular Sciences

MDPI

Table S1. Semiquantitative scoring of GI inflammation for each animal organized by treatment group. GI, gastrointestinal; SUN, sunitinib; RT, radiation therapy.

Treatment Group	Animal ID	PMNs	Lymphs	Plasma cells	Mφ	Giant cells	Cumulative inflammation score	Crypt density/crypt loss	Crypt abscess	Ulceration	Crypt Hyperplasia/ regeneration
**Control**	**1**	0	0	0	0	0	0	0	0	0	0
	**2**	0	0	0	0	0	0	0	0	0	0
	**3**	0	0	0	0	0	0	0	0	0	0
	**4**	0	0	0	0	0	0	0	0	0	0
	**5**	0	0	0	0	0	0	0	0	0	0
**SUN Alone**	**1**	0	0	0	0	0	0	0	0	0	0
	**2**	0	0	0	0	0	0	0	0	0	0
	**3**	0	0	0	0	0	0	0	0	0	0
	**4**	0	0	0	0	0	0	0	0	0	0
	**5**	0	0	0	0	0	0	0	0	0	0
	**6**	0	0	0	0	0	0	0	0	0	0
	**7**	0	0	0	0	0	0	0	0	0	0
**RT Alone**	**1**	1	2	1	1	0	3	3	4	4	3
	**2**	1	1	0	1	0	2	2	4	0	2
	**3**	1	1	0	1	0	1	1	0	0	0
	**4**	1	1	1	1	0	2	2	0	0	2
	**5**	1	1	0	1	0	1	1	0	0	2
	**6**	1	1	0	0	0	1	1	0	0	1
**RT + SUN**	**1**	1	1	0	1	0	2	2	4	0	3
	**2**	1	1	0	1	0	2	1	4	0	3
	**3**	1	1	1	1	0	3	3	4	4	2
	**4**	1	1	0	0	0	1	1	0	0	0
	**5**	1	1	0	1	0	2	2	4	0	3
	**6**	1	1	0	1	0	2	2	4	4	2
	**7**	1	1	0	0	0	1	1	4	0	1

Table S2. Scoring rubric for location of Ki67+ cells as represented by frequency of + cells within the SI villi and extent of + cells around circumference of LI crypt. SI, small intestine; LI, large intestine.

	**Score**		
**Parameter**	**0**	**1**	**2**
Ki67+ Within Villi	Never	Rarely (present in < 10% of villi)	Occasionally (present in 10-30% of villi)
Ki67+ colonic crypt circumference	<50%	>50%	

Table S3. Semiquantitative scoring rubric for gastrointestinal tissues. FOV, field of view.

	Score				
Parameter	0	1	2	3	4
Inflammation	none	1: minimal inflammation with minimal to no separation of crypts (generally focal affecting <10% of mucosa)	2: mild inflammation with mild separation of crypts (generally affecting 11%–25% of mucosa or mild, diffuse inflammatory infiltrates with minimal separation of crypts)	3: moderate inflammation with separation of crypts, with or without focal effacement of crypts (generally affecting 26%–75% of mucosa or moderate, diffuse separation of crypts);	5: diffuse inflammation with marked separation and effacement of crypts (generally affecting >75% of mucosa
Crypt density/crypt loss	normal/none	minimal; decreased by <10%	mild; decreased by 11-25%	moderate; decreased by 25-75%	severe; diffuse loss/effacement of mucosal architecture
Crypt abscesses	absent	-	-	-	present
Ulceration	absent	-	-	-	present
Crypt hyperplasia/regeneration	normal	marked; >10 regenerating crypts per 20x/1.1mm FOV	moderate; 5-10 regenerating crypts per 20x/1.1mm FOV	mild; 1-4 regenerating crypts per 20x/1.1mm FOV	none
